# An Emerging Cardiovascular Disease: Takotsubo Syndrome

**DOI:** 10.1155/2019/6571045

**Published:** 2019-10-30

**Authors:** Sara Moscatelli, Fabrizio Montecucco, Federico Carbone, Alberto Valbusa, Laura Massobrio, Italo Porto, Claudio Brunelli, Gian Marco Rosa

**Affiliations:** ^1^Clinic of Cardiovascular Diseases, University of Genoa, 6 Viale Benedetto XV, 16132 Genoa, Italy; ^2^IRCCS Ospedale Policlinico San Martino Genoa–Italian Cardiovascular Network, 10 Largo Benzi, 16132 Genoa, Italy; ^3^First Clinic of Internal Medicine, Department of Internal Medicine, and Centre of Excellence for Biomedical Research (CEBR), University of Genoa, 6 Viale Benedetto XV, 16132 Genoa, Italy; ^4^First Clinic of Internal Medicine, Department of Internal Medicine, University of Genoa, 6 Viale Benedetto XV, 16132 Genoa, Italy

## Abstract

Takotsubo syndrome (TTS) is a recently identified cardiac disease, which is far from being completely known. The aims of this narrative review are to provide a better understanding of the pathophysiological features of TTS and to update clinical findings in order to improve the management of subjects affected by this syndrome (according to the most recent consensus papers issued by the international scientific societies). We based our search on the material obtained via PubMed up to April 2019. The terms used were “Takotsubo Syndrome and Takotsubo cardiomyopathy” in combination with “heart failure, pathophysiology, complications, diagnosis, and treatment.” TTS is a reversible form of ventricular dysfunction usually characterized by akinesia of the apex in the absence of obstructive coronary artery disease. In its initial phase, TTS may be indistinguishable from AMI and is usually triggered by a sudden emotional/physical stressor which abruptly increases catecholamine levels. However, the mechanisms by which catecholamines or other unidentified molecules can cause myocardial dysfunction is unknown. In-hospital stay may be hampered by various life-threatening complications, while data on long-term survival remain scarce and unclear. Furthermore, TTS may sometimes recur. We believe that TTS is clearly a much more complex condition than previously thought. Much remains to be discovered about its pathophysiologic mechanisms, the role of the link between the heart and brain and that of triggering factors and gender, and the reasons why this syndrome displays different phenotypes and sometimes recurs. Undoubtedly, preliminary evidence from pathophysiological studies (mainly genetic studies) has shown promising advances. However, prospective randomized clinical trials are still needed in order to identify and to tailor the best medical treatments for TTS patients.

## 1. Introduction

In the following paragraphs, we will attempt to update knowledge on both the pathophysiological and clinical aspects of takotsubo syndrome (TTS). We based our search on the material obtained via PubMed up to September 2019. The terms used were “Takotsubo Syndrome and Takotsubo cardiomyopathy” in combination with “heart failure, pathophysiology, complications, diagnosis, and treatment.”

### 1.1. History of the Disease

Several terms (such as happy heart syndrome, broken heart syndrome, and takotsubo cardiomyopathy) have been used to refer to the recently defined takotsubo syndrome (TTS). The first case of TTS was described in Japan (Hiroshima City Hospital) in 1983 [1]. Subsequently, other cases were diagnosed in Japanese patients, leading to the hypothesis that this cardiac disease was typical of Asian populations. More recently, however, a French research group published a case series of TTS in a Caucasian population [2]. TTS gained international attention in the early 2000s [3], when the first diagnostic criteria, including apical dyskinesis/akinesis, absence of obstructive coronary artery disease (CAD) on coronary angiography, new electrocardiographic abnormalities, modest elevation in serum cardiac troponin in the absence of pheochromocytoma, and myocarditis, were published [4]. After the definition of TTS, the first cohorts enrolling around hundred patients allowed description of the heart as indistinguishable from the stunned myocardium [5]. Subsequently, TTS registries were instituted in several countries. In the EU, Spain, Germany, France, and Italy created national registries [6–9]. In Japan, several cohorts and registries were created [10, 11], suggesting the need to collaborate in the creation of an international registry, such as the International Takotsubo (InterTAK) Registry, which was designed as an international, multicenter, prospective and retrospective, and observational study of patients with TTS [12]. Currently, more than 35 international cardiovascular centers in 15 countries contribute to this single registry, including centers in Argentina, Australia, Austria, the Czech Republic, Denmark, Finland, France, Germany, Italy, New Zealand, Poland, Portugal, Switzerland, the United Kingdom, and the United States. The data collected by this registry have enabled new diagnostic criteria and a diagnostic algorithm to be drawn up. The exclusion of a clinically relevant coronary stenosis is to be considered an essential element of this diagnostic algorithm. Moreover, according to the most recent diagnostic criteria, the diagnosis of TTS must also be based on the absence of culprit coronary disease [13, 14] although it is known that TTS may be associated with mild forms of coronary atherosclerosis [15]. TTS is gradually being better investigated and understood, and the number of diagnoses is increasing, the disease being included in the recent 4th universal definition of myocardial infarction by the European Society of Cardiology (ESC) [16]. In Western countries, a female prevalence has been observed, with a female-to-male ratio of 9 : 1 [17]. However, in Japan, men are more affected than women, for unknown reasons [18]. Furthermore, women are more at risk of developing TTS during the menopausal period, suggesting a possible hormonal involvement [19–22]. However, further data are needed in order to confirm this association.

### 1.2. Pathophysiology

Several pathophysiological mechanisms underlying TTS pathophysiology have been hypothesized. The description of takotsubo as a syndrome due to “broken heart” first suggested a critical activity of catecholamines. Indeed, increased circulating levels of these hormones were observed by Wittesten and coworkers in a pilot study comparing patients with takotsubo syndrome with those with acute myocardial infarction (AMI) in Killip class III [3]. The high level of catecholamine seems to be due to hyperactivation of the hypothalamo-pituitary gland-adrenal system in response to an exogenous trigger, which is not always easily recognized. These findings suggested a potential heart-brain interaction in the pathophysiology of TTS. In this regard, the limbic network, comprising the insula, amygdala, cingulate cortex, and hippocampus, and the autonomic nervous system appear to suffer some degree of dysfunction in patients with TTS in comparison with healthy controls [23]. Moreover, TTS recurs in patients with ictus or aneurysmal subarachnoid hemorrhage or epilepsy, which affect the abovementioned nervous regions [24–27]. It has been discussed whether the transient, regional left ventricular dysfunction associated with several neurological disorders shares the same pathophysiology with TTS without a clear neurological disease and whether it is appropriate to include these patients in a single category of stress cardiomyopathy. This issue requires additional evidence from both clinical and basic research studies.

The supposed mechanisms by which catecholamine excess can aggravate myocardial stunning remain to be clarified. The spasm of epicardial arteries and the acute worsening of endothelial dysfunction have been investigated without definite conclusions being reached [28]. Another hypothesis focused on microcirculation dysfunction, which may be inferred from the presence of reduced TIMI flow, and also on endothelial cell necrosis in myocardial biopsies [29, 30]. In addition, intravenous administration of adenosine has been shown to transiently improve myocardial perfusion, wall motion score index, and left ventricular ejection fraction (LVEF) in TTS, suggesting that intense microvascular constriction could play a major pathophysiological role [31].

Transient LV dysfunction in TTS could also result from detrimental catecholamine-induced activity on cardiomyocytes. It has been hypothesized that a high level of epinephrine signaling takes place through the pleiotropic 2-adrenergic receptor (2AR) from canonical stimulatory G-protein-activated cardiostimulant to inhibitory G-protein-activated cardiodepressant pathways. Because the majority of B2 receptors are located on the LV apex, altered intracellular signaling may be the cause of the apical ballooning appearance [32].

Finally, it has been suggested that some anatomic variants of coronary vessels may also play a role [33]. Indeed, Arcari and coworkers [34] demonstrated that coronary artery tortuosity and a long, recurrent wrap-around the left anterior descending artery might act as a potential pathogenic substrate in TTS. It has been hypothesized that, in the presence of sympathetic overdrive, the subsequent tachycardia, with decreased diastolic time and increased contractility, could aggravate the hemodynamic effect of these anatomic variants (also including myocardial bridges), thus facilitating the onset of the peculiar wall motion abnormalities of TTS. Taking into account both the presence of familial cases and the fact that only a small percentage of women who undergo psychological stress develop TTS, the existence of a genetic predisposition may be hypothesized [35]. However, genetic studies have yielded conflicting results concerning the potential association between TTS and polymorphisms of adrenergic receptors [36, 37]. In the largest genetic study, Eitel et al. [36] detected 68 promising candidate loci; unfortunately, none of these reached genome-wide significance. Nevertheless, this result may be considered an important step towards further insights into a potential genetic predisposition for the development of TTS.

Some genetic variants probably interact with the environment, and certain predisposed individuals may develop TTS more easily. In accordance with all these data, since many factors have been called into play in the pathophysiology of TTS, the ultimate etiology of the disease must be regarded as multifactorial.

### 1.3. Clinical, Laboratory, and Imaging Presentation

Like an AMI, TTS can present with acute chest pain, dyspnea, and syncope [38]. On the contrary, patients may sometimes be completely asymptomatic, and TTS is discovered because of ECG changes or imaging alterations [39]. Moreover, a minority of TTS patients may suffer symptoms due to complications such as heart failure, pulmonary edema, stroke, cardiogenic shock, or cardiac arrest [38].

Historically, TTS has been related to a preceding emotional stressor. However, international takotsubo registries have revealed that, in 30% of patients, no trigger event can be identified, and that both emotional trauma and physical stressors can precede TTS [38, 40, 41]. In addition, in men, physical traumas seem to be more frequent than emotional triggers, whereas, in women, emotional stressors are more frequently detected [4, 9]. As TTS may recur, it is of paramount importance to identify specific triggers. In an interesting paper, Finsterer and coworkers discussed the matter of what triggers TTS in cases without obvious triggers and underlined the concept that triggers have to be both identified and defused [42]. In order to detect the stressor stimulus triggering TTS, it is mandatory to scrutinize the individual's history for such events. However, patients may be sometimes reluctant to talk about stressful events; if so, relatives or close friends can be also consulted [42]. Finally, the presence of a preexisting subclinical cardiomyopathy may be a copathophysiological factor; any such condition must be identified and monitored, as it can worsen the outcome. Morner and colleagues described the case of heart failure with echocardiographic findings of a large aneurism of the left ventricle, which occurred after gastrointestinal surgery in a patient with hypertrophic obstructive cardiomyopathy [43]. The authors suggested that a superimposed TTS may account for the occurrence of heart failure symptoms and signs in a portion of cases with preexisting myocardial disease. Unfortunately, in this case, the lack of serial echocardiograms did not allow this to be ascertained. Other authors have described cases of TTS occurring in the context of either hypo- or hyperthyroidism, which may be associated with coronary microcirculatory dysfunction and abnormal myocardial expression of adrenergic receptors or with hyperactivation of the adrenergic system, which might serve as a substrate for the myocardial stunning [44, 45]. With regard to EKG alterations, TTS often presents abnormalities, the most common being ST-segment elevation, which is found in 44% of cases and mimics an acute myocardial infarction. In addition, T-wave inversion may sometimes also occur [38]. Despite some overlap between anterior STEMI and TTS, some EKG criteria have been proposed in order to distinguish between these two diseases [46, 47]. In TTS, ST-segment elevation is mainly localized in V2–V5 leads and in II and aVR. By contrast, in anterior STEMI, ST-segment elevation is detectable on precordial leads V1–V4 and limb leads I and aVL. Another criterion is the absence of reciprocal changes in inferior leads in TTS [46]. Furthermore, ST-segment elevation occurs more frequently in aVR and less frequently in V1, which is generally involved in anterior STEMI. ST-segment elevation that is limited to the inferior leads (II, III, and aVF) is distinctly uncommon in TTS [46–48].

Another EKG feature of TTS is the absence of Q waves. Furthermore, ST-segment depression is uncommon in TTS, occurring in less than 10% of patients, while it occurs in more than 30% of other acute coronary syndrome (ACS) patients [38]. In addition, similarly to ACS, in TTS, the EKG displays a temporal evolution, typically with a resolution of initial ST-segment elevation (if present), followed by progressive T-wave inversion and QT interval prolongation, which may be considered another characteristic of TTS [49, 50].

The reversible nature of TTS has given rise to the belief that it is a benign disease. However, the registries that have been created in recent years have revealed that TTS has a similar rate of in-hospital complications to AMI [38, 39]. TTS patients need to be monitored, owing to the various in-hospital complications that may arise as a result of electrical and hemodynamic instability, such as cardiac arrhythmias, cardiogenic shock, ventricular thrombus, pulmonary edema, ventricular septal defect, and free-wall rupture. Furthermore, male patients have up to threefold higher rate of death and major adverse cardiac and cerebrovascular events, contributing to a higher mortality rate [39].

In-hospital complications may include various forms of arrhythmia. Patients with TTS often have sinus tachycardia although sinus bradycardia or other bradyarrhythmias may also occur. Moreover, multiple atrial and ventricular premature beats, sinus node dysfunction (SND), asystole, paroxysmal supraventricular tachycardia (PSVT), atrial fibrillation (AF), nonsustained and sustained ventricular tachycardia (VT) (both monomorphic and polymorphic torsades de pointes (TdP)), ventricular fibrillation (VF), ventricular asystole, pulse-less electrical activity, sudden cardiac death (SCD), and right and left bundle branch blocks can be detected [51].

Atrial fibrillation (AF) is present in approximately 4.7% of patients with TTS, and permanent oral anticoagulation should be considered [39]. Cardiac magnetic resonance imaging has revealed an association between transient myocardial edema, as evidenced by T2-weighted sequences and dynamic T-wave inversion and QT prolongation [52–54]. Finally, arterial thromboembolism constitutes the second most frequent complication of TTS [55–57]. In a recent study, Abanador-Kamper and coworkers found that stroke in TTS had an event rate of 2.8% after 30 days and 4.2% after 12 months [57]. Francesco Santoro and colleagues identified some characteristics linked to thromboembolism and created a therapeutic algorithm to potentially improve the clinical management [58]. For instance, in the case of an apical ballooning pattern and increased admission levels of troponin-I (>10 ng/mL), oral anticoagulation must be considered, while in the case of midventricular/basal ballooning or apical ballooning associated with troponin-I levels <10 ng/mL, oral anticoagulation should not be initiated. A simple combination of echocardiographic parameters (apical ballooning pattern), EKG data (ST-elevation on admission and persisting after 72 h), and laboratory values (troponin serum levels) could be useful to the appropriate therapeutic management of oral anticoagulation in TTS [59].

On echocardiography, circumferential abnormalities of anterior, inferior, and lateral ventricular wall contraction extending beyond a single epicardial vascular territory can be considered a hallmark of TTS [59]. Also, a hyperkinetic basal segment is currently described in the most common form of TTS. Other contraction patterns may be present during the acute phase of TTS, such as midventricular akinesia [60], basal segment akinesia with midventricular and apical sparing [61], and the focal variant, characterized by focal wall motion abnormalities [62].

Furthermore, echocardiographic parameters may be useful predictors of adverse outcomes. Indeed, adverse outcomes can be predicted by low left ventricular ejection fraction, increased LV filling pressure, and moderate-to-severe mitral regurgitation at 4–6 weeks [63]. Wall motion abnormalities tend to revert in 4–8 weeks. Another important diagnostic tool is cardiac magnetic resonance (CMR), which is usually performed in a subacute phase [64]. As this technique has higher resolution, it can better identify wall motion alterations and complications; it also offers a unique combination of safety, detailed anatomical visualization, and tissue characterization data [65]. The most recent diagnostic criteria of TTS highlight the essential role of CMR, not only for morphological characterization but also for the exclusion of other entities that otherwise would not be ruled out, particularly myocarditis and myocardial infarction with nonobstructive coronary arteries (MINOCA). In an article published in JAMA in 2011, CMR diagnostic criteria for TTS were established [66]: firstly, the presence of wall motion alterations is necessary; secondly, the dysfunctional segments must show edema on T2W sequences, and finally, late gadolinium enhancement (LGE) should not be present. The absence of LGE suggests that there is no fibrosis or increased extracellular space. However, in 9% of cases, some spots of fibrosis have been found, and these are related with the worst prognosis [67].

CMR can be useful in distinguishing the focal forms of TTS from an ACS or myocarditis, in which wall motion alterations do not extend beyond the distribution of a single coronary territory; however, even this tool may sometimes fail to obtain a differential diagnosis. Undoubtedly, coronary angiography and ventriculography are essential to the differential diagnosis from ACS [68]. Nuclear medicine techniques may be usefully used for the diagnosis of TTS and have increased our knowledge of the pathophysiological mechanisms. Indeed, they have demonstrated that dysfunctioning segments present a pattern of severe denervation and metabolic glucose uptake, which ameliorates and even normalizes, during follow-up as a result of the improvement in myocardial function. Studies based on positron emission tomography and myocardial SPECT have shown a discrepancy between normal perfusion and reduced glucose utilization in TTS, commonly known as “inverse flow metabolism mismatch” [69, 70].

In the acute phase, TTS may be clinically indistinguishable from acute MI but, by means of positron emission tomography, it has been successfully distinguished from acute coronary artery disease, thereby also offering a new pathophysiological explanation for this particular syndrome. Apical ballooning syndrome may be considered a disorder at the cellular level rather than a structural contractile disease of the myocardium, as a transient decrease of glucose metabolism might be related to a coronary microcirculation impairment, followed by prolonged myocardial stunning [71].

Serum troponin is usually positive in the acute phase of TTS, but its values tend to be low in comparison with the extension of myocardial dysfunction. It is commonly believed that troponin levels in AMI are higher than in TTS, though the InterTAK Registry has shown no difference between the two pathologies [63]. On the contrary, the hs-TnT/CKMB ratio could be helpful in differential diagnoses, in that it has been found to be significantly higher in TTS patients than in NSTEMI and STEMI [72].

During the course of TTS, serum levels of NT-proBNP also increase. Nef and colleagues found that serum levels of NT-proBNP on admission were correlated to the severity of post-TTS complications during hospitalization [73]. The higher serum levels of NT-proBNP were on admission, and more clinical complications were present, i.e., pulmonary edema and malignant ventricular arrhythmia. Furthermore, Frochlich and coworkers [74] have suggested that TTS presents with specific NT-proBNP/myoglobin and NT-proBNP/troponin T ratios that can be differentiated from those observed in NSTEMI and STEMI.

### 1.4. Diagnostic Criteria

In recent years, there have been several attempts to create effective diagnostic criteria for TTS. The most widely used since 2018 have been the Mayo Clinic Diagnostic Criteria [4, 75]. The European Society of Cardiology (ESC) has also established the International Takotsubo Diagnostic Criteria (InterTAK Diagnostic Criteria) [13], which implement a diagnostic algorithm and assign a score to TTS. The InterTAK Diagnostic Score considers the following variables and gives each one a specific score [13, 14]: female sex; emotional stress and physical stress; no ST depression; psychiatric disorders; neurologic disorders; and QTc prolongation. If the score is ≤70 points, the probability of having TTS is intermediate or low, whereas a score ≥70 indicates a high probability (Table 1). The patients with a low probability of TTS and a suspicion of ACS should undergo coronary angiography and left ventriculography, while in patients with a high score, transthoracic echocardiography (TTE) should be performed. Wall motion abnormalities on TTE help to decide the next step; if the typical presentation with circumferential ballooning pattern is absent, coronary angiography with left ventriculography is the choice. In normal coronaries and typical ballooning, myocarditis must be excluded. The algorithm includes some clinical markers of myocarditis called red flags (signs and symptoms of viral infection, elevated ESR and/or PCR, and pericardial effusion); if these are present, CMR is the only tool that can help.

## 2. Treatment

The management of TTS is based on both clinical experience and Expert Consensus, as no prospective randomized clinical trials have been carried out.  Table 2 summarizes the main drugs that are recommended to treat TTS.

### 2.1. Pharmacological Treatments

Owing to the similarities in clinical presentation of AMI and TTS in the earliest phase, the therapy is the same: antithrombotic, heart failure drugs, and aggressive statin therapy should be undertaken [39, 76–78]. Once the diagnosis has been established, if concomitant atherosclerosis is present, statin and aspirin are recommended. As high levels of circulating catecholamines have been detected in TTS, it is not recommended that epinephrine and norepinephrine be used as inotropic support; rather, levosimendan, which is a Ca2+ sensitizer, could fulfill this function [79]. Its use, however, is limited to those patients with impaired systolic function, without left ventricular outflow tract obstruction and with systolic blood pressure >90 mmHg. Levosimendan should be infused for 24 h at a dose of 0.1 *μ*g/kg/min, without loading dose. During infusion, the patient should be hemodynamically and EKG monitored for the risk of hypotension and arrhythmias [79]. The effects of catecholaminergic storm during TTS could be attenuated by the use of beta-blockers. In an animal trial, both alpha- and beta-blockers were used with positive results, but more data are required before these strategies can be used in humans [80]. Furthermore, beta-blockers can be used safely in 20% of TTS patients with left ventricular outflow tract obstruction (LVOTO), as they seem able to reduce the intra-aortic gradient [81].

In addition, other studies have found an increasing concentration of beta-2 receptors from the base to the apex of the LV and a higher concentration of beta-1 in the basal left ventricular segments, suggesting the use of selective beta-1 beta-blockers [82]. Owing to the fact that TTS patients sometimes present psychiatric or neurologic pathologies and so have prolonged QTc (>500 msec) and increased risk of TDP [83], we must pay particular attention when prescribing beta-blockers for these subjects [83]. For this reason, they should be monitored hemodynamically and by means of EKG, and short-acting BB should be preferred [84]. Regarding the long-term use of BBs, even though they can counterbalance the action of catecholamines, no studies have demonstrated a reduction in recurrences [85, 86]. Randomized controlled trials need to be conducted in order to clarify the real efficacy of BBs. This category of drug seems capable of promoting the recovery of the LV, reducing not only mortality at 1 year but also the number of recurrences [38]. By contrast, BBs have not shown these potentialities [85] even though the combination of BBs and ACI/ARBS appears to be connected with lower rates of recurrences [86, 87]. Diuretics can be used to reduce edema, while nitrates are useful to ameliorate LV and RV filling pressures and after-load; however, nitrates should be avoided in the case of LVOTO [78].

### 2.2. Mechanical Cardiac Support (MCS)

If patients present with cardiogenic shock, the use of mechanical circulatory support as a bridge to recovery therapy can be considered. The IABP has not yielded positive results, as it can raise the transaortic gradient [88, 89]. The TandemHeart is theoretically useful, but its efficacy has not been documented in the current literature [90]. The ECMO reduces preload and increases end-organ perfusion at the cost of increased after-load, causing a possible rise in the transaortic gradient and worsening of mitral regurgitation [91]. Impella appears to provide the most effective support, as it has been seen to overcome LVOTO and reduce mitral regurgitation [92, 93].

### 2.3. Prognosis and Survival

Available data on long-term survival after TTS are limited. In 2007, Elesber and coworkers [94] reported that long-term mortality did not differ between patients with TTS and an age-matched population. Conversely, Sharkey et al. [95] found that all-cause mortality during follow-up exceeded that of a matched general population, with most deaths occurring in the first year. More recently, it has been reported that long-term mortality in patients with TTS is similar to that of patients with CAD. Specifically, TTS patient data from the Swedish Angiography and Angioplasty Registry (SCAAR) from 2009 to 2013 were compared with data from patients with and without CAD and demonstrated that mortality rates for TTS were worse than in patients without CAD and comparable to those of patients with CAD [96]. In the largest TTS registry to date, death rates are estimated to be 5.6% and the rate of MACCE 9.9% per patient-year, suggesting that TTS is not a benign disease [38]. A study conducted by Ghadri et al. found that patients with the typical TTS type have a comparable outcome to patients presenting with the atypical type, even after adjustment for confounders, suggesting that both patient groups should be equally monitored in the long term [62]. On the contrary, 1-year mortality differs between the two groups, as it is driven by clinical factors, including atrial fibrillation, LVEF <45% on admission, and neurological disorders, rather than by the TTS type [62]. In a smaller study, predictive factors of long-term mortality in TTS were male sex, Killip class III/IV, and diabetes mellitus [97]. The prognostic role of diabetes mellitus is controversial, as it is postulated that it may exert a protective effect in TTS, given that the prevalence of diabetes mellitus in TTS is lower than expected for an age- and sex-matched population [98]. Some studies, though limited by their population size and experimental design, have suggested that patients with diabetes mellitus have a more favorable in-hospital and 1-year outcome [99, 100]. Recent reports have suggested a high rate of noncardiovascular long-term mortality in TTS, which could be ascribed to malignancies [100, 101]. Another possible complication is that of recurrences. Recurrence may take place after as little as 3 weeks, or as late as several years, after the initial event [39]. Furthermore, in recurrent TTS, the LV ballooning pattern may differ; for example, apical ballooning may occur during the initial event and midventricular ballooning during the second event [102]. The recurrence rate of TTS has been reported to be up to 12% over a mean follow-up of four years and may display a variable pattern [102]. Finally, we should remember that the prognosis of TTS may differ according to the type of trigger. When TTS is induced by a physical event, rather than by emotional stress, the prognosis is worse. Indeed, in the presence of physical stressors, a fourfold higher mortality risk has been found, while in the presence of unidentifiable triggers, the risk is doubled [103].

## 3. Conclusions and Perspectives

TTS must be considered an intriguing and fascinating disease, owing to the fact that it is a far more diverse condition than previously thought and that the pathophysiologic mechanisms are not completely understood. Indeed, although the correlation of TTS with a surge in catecholamines has been clearly demonstrated, there is no established pathophysiological mechanism; however, several promising hypotheses have been put forward. Undoubtedly, a link between the brain and heart seems to play a key role. The predominance of postmenopausal women in TTS suggests that sex hormones and the endocrine system may play a role. However, much remains to be discovered about TTS and its pathophysiology. Furthermore, many issues remain to be investigated, such as the role of triggering factors and gender, why TTS displays different phenotypes, and which patients are most prone to developing TTS or its recurrences. To answer these questions, additional studies are needed. While TTS was formerly considered a benign disease, recent studies have shown that it presents in-hospital complications and mortality that are comparable to those of ACS. However, data on long-term survival are scarce and somewhat unclear. Randomized, prospective, multicenter trials involving larger numbers of patients must be conducted in order both to identify effective evidence-based therapies, in the in-hospital phase and the longer term, and to prevent recurrences.

## Figures and Tables

**Table 1:** Diagnostic criteria used to distinguish takotsubo syndrome (TTS) from acute coronary syndrome (ACS).

InterTak diagnostic score	
Female sex	25 points
Emotional stress	24 points
Physical stress	13 points
No ST depression	12 points
Psychiatric disorders	11 points
Neurologic disorders	9 points
QTc prolongation	6 points
Score >70 points: high probability of TTS	
Score ≤70 points: low/intermediate probability of TTS	

**Table 2:** Drugs recommended to treat takotsubo syndrome (TTS).

Drugs	Main indication
Levosimendan	Ejection fraction <55%, but not when left ventricular outflow tract obstruction (LVOTO) arterial pressure >90 mmHg
Beta-blockers	QTc <500 msec LVOTO
ACE inhibitors/ARBs	To reduce the rate of recurrence
Diuretics	To reduce edema
Nitrates	No if LVOTO
